# Book Reviews

**Published:** 1901-02

**Authors:** 


### BOOK REVIEWS, PAMPHLETS, EXCHANGES.

### BOOK REVIEWS.

[Contributions solicited for review.]

DISEASES OF WOMEN. A TREATISE ON THE PRINCIPLES AND PRACTICE OF GYNECOLOGY, for students and practitioners. By E. C. Dudley, A.M.,M.D., Chicago. Second Edition. Revised and enlarged, with 453 illustrations, of which 47 are in colors, and 8 full-page plates in colors and monocrome. Lea Brothers & Co., Philadelphia and New York.

Besides the original subject matter of the first edition, which has been carefully revised, the second edition contains additional chapters on premature menstruation, protracted menstruation, amenorrhea and scanty menstruation, menorrhagia, and metrarrhagia, dysmenorhea and sterility. Many new illustrations have been added. The subjects are grouped on the basis of etiological and pathological relation, an arrangement which at once commends itself to the convenience and comprehension of the reader. The work is compact, clear and explicit, and the second edition will doubtless add to the popularity already secured by the first issue.

---

PANAMA AND THE SIERRAS: A DOCTOR'S WANDER-DAYS. By G. Frank Lydston, M.D. Illustrated from the author's original photographs. The Riverton Press, Chicago. 1900.

Dr. Lydston has already won for himself an enviable position in general literature. His "Tales of a Talkative Doctor" will be remembered with pleasure by many readers. "Panama and the Sierras" is an account of Dr. Lydston's travels through tropical America and California, and is a most entertaining and attractive narrative. His style is bright and fluent. The volume abounds in humor, and descriptions of scenery and of men which show the author's appreciative love of nature and his shrewd observation of human character. No one will read the book without envying Dr. Lydston for his pleasant experience, or without at the same time being grateful to him for allowing one to enjoy it with him without having to endure the annoyances and minor hardships incident to his travels.

---

URINARY DIAGNOSIS AND TREATMENT. By J. W. Wainwright, M.D., Member of the American Medical Association, New York State Medical Association, New York County Medical Association, etc. Illustrated with numerous engravings and colored plates. Pages, 140. Price, $1.00 net.

This book gives not only all the usual methods or urinary examination, but introduces also a new feature in works of this character, viz: A discussion of the clinical significance of the urinary findings and their practical application in treating the diseases of which they are symptomatic.

Among the subjects discussed are the following: Composition and Physical Character of the Urine; Normal Constituents of Urine; Abnormal Constituents; the Microscope and Microscopical Technique; Qualitative Analysis of Urinary Calculi; Bright's Disease, Diabetes, Gout and other Conditions and Their Treatment; Favorite Prescriptions.

---

DUANE'S MEDICAL DICTIONARY. A Dictionary of Medicine and the Allied Sciences. By Alexander Duane, M.D., Assistant Surgeon to the New York Ophthalmic and Aural Institute. Lea Brothers & Co., Philadelphia and New York.

The need of good medical dictionaries in educating the profession to greater accuracy in the use of words and the definition of terms is most apparent. One will often find on consulting a dictionary, that many words which he has been employing have been more or less misapprehended as to their exact meaning. Medical terminology is notably arbitrary and confusing, and any book tending to an easier acquirement of a knowledge of it is welcome.

Duane's Dictionary offers much in this direction. It includes also pharmacy, dentistry and veterinary medicine, and has, in addition, brief notices of the principal diseases, drugs and their uses. The book is a handsome volume and in every way complete and convenient.

RUDIMENTS OF MODERN MEDICAL ELECTRICITY. Arranged in the form of Questions and Answers. Prepared especially for Students of Medicine. By S. H. Monell, M.D., New York. 12mo. About 150 pp. Price, Cloth, $1.00. New York: Edward R. Pelton, No. 19 East Sixteenth St. 1900.

In view of the surprising amount of ignorance of the profession in general on the subject of electricity as applied to medical uses, a book of this character is especially needed. It sets forth in the simplest form—that of questions and answers—the elemental facts of electro-therapeutics. Medical electricity is defined, the various currents described, and the apparatus illustrated and, finally, indications for their use are given. The book can be commended as a plain and simple primer applicable not only to the student, but to the practitioner, who should be corrected of his haphazard methods of administering an important therapeutic agent.

---

KING'S AMERICAN DISPENSATORY. By Harvey Wickes Felter, M.D., and John Uri Lloyd, Phr.M. Ph.D. Entirely rewritten and enlarged. Eighteenth Edition. Third Revision. In two volumes. Vol. II., G to Z. Cincinnati. The Ohio Valley Co., 317–321 Race St. 1900.

This book is designed primarily to meet the needs of the Eclectic School of Medicine, but its value extends far beyond such meager limitations. It is a wonderful work on medical botany, and the therapeutic uses of indiginous American flora. The vast amount of time and labor expended upon it, and the splendid result, make King's Dispensatory not only a classic but an honor to the nation. No one can be insensible to the merit of the book, and every practitioner of medicine should possess it.

---

TRANSACTIONS OF THE AMERICAN ORTHOPEDIC ASSOCIATION, Fourteenth Sesson, held at Washington, D. C., May 1, 2, 3, 1900. Vol. XIII. Published by the Association. Philadelphia. 1900.

This volume contains a number of fine contributions to orthopedics, notably a paper by A. M. Phelps, on lateral curvature of the spine. Other contributors are John Ridlon, Goldthwaite,

Davis, Taylor, Bradford, Tubby, V. P. Gibney, Lovett, DeForest Willard, besides other well known orthopedic surgeons. The volume is well illustrated, and the letter press and binding are excellent.

---

A BOOK OF DETACHABLE DIET LISTS. For albuminuria, anemia, and debility, constipation, diabetes, diarrhea, dyspepsia, fevers, gout or uric acid diathesis, obesity, tuberculosis and a sick-room dietary. Compiled by Jerome B. Thomas, Jr., A.B., M.D., Instructor in Materia Medica Long Island College Hospital; Assistant Bacteriologist to Haagland Laboratory. Second edition; revised. Published by W. B. Saunders, 925 Walnut Street, Philadelphia. 1900.

This book is arranged with a view to regulating the diet of the sick and convalescent by checking a dietary from a detachable list. The various articles of food are listed, as also the diseases and conditions in which they are indicated or admissible. There are also to be found a large number of formulæ for preparing various aliments, and instructions for the preparation of food in general with suggestions as to administration. The book will prove of great assistance to physicians and those who have charge of the sick.

---

DISEASES OF THE HEART; THEIR DIAGNOSIS AND TREATMENT. By Albert Abrams, A.M., M.D., San Francisco, Consulting Physician for Diseases of the Chest Mt. Zion Hospital and the French Hospital. Illustrated. Pages, 172. Price, $1.00 net.

In this book the author discusses the subject of diseases of the heart entirely from a practical aspect. His most noteworthy researches in methods of diagnosis are here recorded for the first time in collected form, and the latest and most practical methods of treatment given in detial.

